# The perverse impact of external reference pricing (ERP): a comparison of orphan drugs affordability in 12 European countries. A call for policy change

**DOI:** 10.1080/20016689.2017.1369817

**Published:** 2017-09-03

**Authors:** K. E. Young, I. Soussi, M. Toumi

**Affiliations:** ^a^ Pricing, Reimbursement, and Market Access, Creativ-Ceutical, Paris, France; ^b^ Pricing, Reimbursement, and Market Access, Creativ-Ceutical, Tunis, Tunisia; ^c^ Department of Public Health, Aix-Marseille Université, Marseille, France

**Keywords:** Rare diseases, orphan drugs, pricing, differential pricing, external reference pricing, treatment cost, relative costs, affordability, ability to pay, Europe

## Abstract

**Objective**: The study compared the relative cost differences of similar orphan drugs among high and low GDP countries in Europe: Bulgaria, France, Germany, Greece, Hungary, Italy, Norway, Poland, Romania, Spain, Sweden, UK.

**Methods**: Annual treatment costs per patient were calculated. Relative costs were computed by dividing the costs by each economic parameter: nominal GDP per capita, GDP in PPP per capita, % GDP contributed by the government, government budget per inhabitant, % GDP spent on healthcare, % GDP spent on pharmaceuticals, and average annual salary. An international comparison of the relative costs was done using UK as the reference country and results were analysed descriptively.

**Results**: 120 orphan drugs were included. The median annual costs of orphan drugs in all countries varied minimally (cost ratios: 0.87 to 1.08). When the costs were adjusted using GDP per capita, the EU-5 and Nordic countries maintained minimal difference in median cost. However, the lower GDP countries showed three to six times higher relative costs. The same pattern was evident when costs were adjusted using the other economic parameters.

**Conclusion**: When the country’s ability to pay is taken into consideration, lower GDP countries pay relatively higher costs for similarly available orphan drugs in Europe.

## Introduction

Rare diseases are serious conditions which are defined in the European Union (EU) as life-threatening or chronically debilitating conditions with a prevalence of no more than five in 10,000 people [1]. In the UK, the National Institute for Health and Care Excellence (NICE) in England and Wales has defined ultra-rare diseases as diseases affecting fewer than 1000 patients [2]. Orphan drugs are medicines intended for the prevention or treatment of rare diseases.

Rare diseases are usually severe conditions with no or limited choice of therapeutic options. In 2007, the European Medicines Agency (EMA) estimated that there are 5000–8000 rare diseases affecting 6–8% of the total EU population, amounting to 27 million to 36 million people in the EU [1]. Current figures are assumed to be higher, with the same report documenting that five new diseases are described in the medical literature every week. It is estimated that only 1% are currently covered by approved treatments in the EU [3].

Orphan drugs prices, which are higher than non-orphan drugs, have been continuously discussed despite the high level of unmet needs associated with rare diseases. A 2011 budget impact study in 18 European countries [4] showed that the annual treatment cost per patient of commercially available orphan drugs varied between €1251 and €407,631 with a median of €32,242. Orphan drugs’ share of the total pharmaceutical market was predicted to peak from 3.3% in 2010 to 4–5% in 2020 [4].

Although orphan designation is at the European level and marketing authorisation can be both at the European and national level, pricing and reimbursement are on a national level often driven by health technology assessments (HTA) outcomes and a variable impact from external reference pricing (ERP) [5]. Thus, price [6] and access to orphan drugs vary among countries in the EU [7–10].

We assessed the price differences of orphan drugs among European countries in our previous study [11] by comparing the annual treatment cost per patient of similarly available drugs in seven countries (France, Germany, Italy, Norway, Spain, Sweden, and the UK). Our results showed that although the mean annual treatment costs of orphan drugs varied minimally among the seven countries, there were wide ranges in the costs which indicate price heterogeneity across and within all the seven countries. Given the differences in eachcountry’s ability and willingness to pay for healthcare, we recommended that further analysis is needed to compare relative costs, adjusted using economic parameters, to validate the pattern of price differences and access among European countries.

This current study compared economic parameters-adjusted annual treatment costs of orphan drugs covering an exhaustive sample of available products in low and high gross domestic product (GDP) countries in Europe. Twelve countries were included in the study, sampling a large EU population representation (83% of the EU population [12]) of a variety of geography and country sizes (east/west, north/south, and large/small). All the countries included provide orphan drugs to their citizens via public health insurance although the scope, breadth, height, and depth of funding may vary.

## Methods

This study compared the price differences of orphan drugs among countries by comparing the annual treatment cost per patient adjusted by nominal GDP per capita, GDP in Purchasing Power Parity (PPP) per capita, % GDP contributed by the government, government budget per inhabitant, % GDP spent on healthcare, % GDP spent on pharmaceuticals, and average annual salary of similarly available orphan drugs in low and high GDP countries in Europe: Bulgaria, France, Germany, Greece, Hungary, Italy, Norway, Poland, Romania, Spain, Sweden, and the UK.

### Extraction of relevant data

Orphan drugs granted market authorisation up to 13 June 2016, including drugs with expired or withdrawn orphan drug designations, were extracted for analysis.

The IHS POLI database [13] was the primary source of price data. For drugs with no available prices in POLI, available country-specific price databases were used: database of drugs and tariffs (Ameli) [14] for France; British National Formulary (BNF) [15] for the UK; and Farmadati Compendio Farmaceutico Telematico database [16] for Italy. The earliest price was used for cost calculation as we are interested in the prices at launch and drug prices change over time. An exception to this was when the BNF was used, where current prices were extracted because price history was not available. Prices in local currencies for Bulgaria, Hungary, Norway, Poland, Romania, Sweden, and the UK were converted to euros using 2015 annual average exchange rates.

Economic parameters including nominal GDP per capita, GDP in PPP per capita, GDP share contributed by the government (%), government budget per inhabitant, GDP share spent on healthcare (%), GDP share spent on pharmaceuticals (%), and average annual salary for all 12 countries were retrieved from the Organisation for Economic Co-operation and Development (OECD) database [17] or other official sites when not available in the OECD [18–20]. Figures in US dollars and local currencies were converted to euros using 2015 exchange rates.

### Annual treatment cost calculation

We calculated the annual treatment cost per patient in each country for each orphan drug based on the posology described in the Summary of Product Characteristics (SmPC). Across all 12 countries, the indication and posology are the same. There are differences in the preparation and formulation of drugs across countries but these are minor. As much as possible, the same formulation and preparation per product were used in all countries for comparability.

As dosing of orphan drug treatments may vary according to patient age, weight, disease severity, patient needs, disease progression, or disease complications, assumptions were used for the dose and cost computations.

- Average drug dose for an adult was used unless specifically indicated for use in children. For drugs indicated for both adults and paediatric populations, the pivotal studies described in the European Public Assessment Report (EPAR) were consulted for the average age of the population included in clinical trials and dosage and cost computation were done for this specific average patient. For weight-adjusted and Body Surface Area (BSA)-adjusted treatments, the average weight of an adult was set at 70 kg and the average body surface area was set at 1.73m^2^. Standard average values for other age intervals were also used [21].
- If the dose is adjustable based on performance results or an average dose was given, information regarding the average treatment duration and dosage from the EPAR and pivotal studies was used. In the same manner, for cycle-based treatments where the number of cycles varies, the mean number of cycles in the pivotal trials was assumed.
- Treatment duration of 365 days was assumed. For drugs used for less than a year, the costs of the total treatment course were analysed as annual costs.
- For treatments administered as injection or infusion, the closest full vial size was used. The EPAR was consulted if vials can be stored once opened or should be used within the day. Vial wastage in this sense was taken into consideration.
- If there was an unfinished pack at the end of the year or at the end of a treatment cycle, only a proportion of the price of that pack was accounted for.

We adjusted the annual treatment cost for each orphan drug by dividing the costs by each economic parameter described above, generating eight types of relative costs for each drug per country.

### Inter-country comparison of adjusted treatment costs of similarly available drugs

Out of the 12 countries, the country with the most orphan drugs with available prices was set as the reference country. The UK had the most available price data hence was used as the reference country for subsequent analyses.

For each orphan drug, the adjusted treatment cost in the comparator country was divided by that of the UK and the ratios were computed. The obtained cost ratios represented cost differences between the two countries. With the UK set as 1, a ratio of >1 means that the orphan drug is more expensive than the UK counterpart and a ratio of <1 means the orphan drug is cheaper than the UK counterpart. For each country, the obtained ratios were averaged and median, minimum, and maximum values were identified. Analyses were done for each of the seven economic parameters.

## Results

Ninety-five authorised orphan drugs were identified in the EMA website between 2002 and 2016 and were complemented with 25 drugs with expired or withdrawn orphan drug designations using Creativ-Ceutical’s unpublished database for a total of 120 drugs (Appendix A in the supplementary file).

Commercial availability of orphan drugs varied in the 12 countries and not all commercially available orphan drugs had publicly available prices for analysis. The UK had the largest number of orphan drugs with available price (N = 94). When the UK reference list was compared to the orphan drugs available in the other 11 countries, the sample sizes for analysis were as follows: UK-Italy 76, UK-France 65, UK-Germany 62, UK-Norway 61, UK-Greece 60, UK-Romania 48, UK-Spain 40, UK-Bulgaria 38, UK-Sweden 35, UK-Poland 31, and UK-Hungary 21.

**Figure 1.**
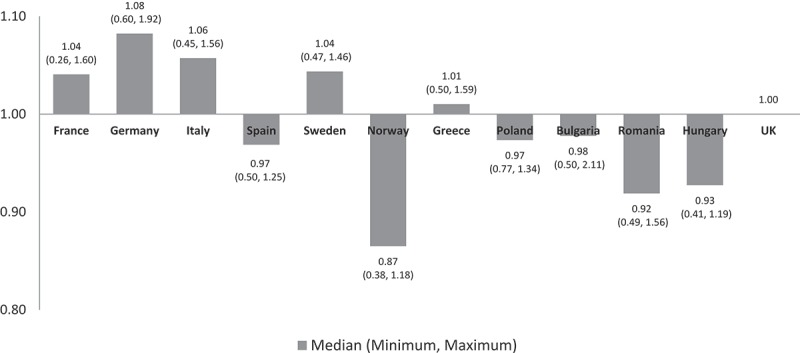
Unadjusted annual cost ratios using the UK as a reference.

The median values of the non-adjusted annual costs varied minimally in all countries except Norway which had a slightly lower cost ratio of 0.87 reflecting 13% lower median costs than the UK (Figure 1).

**Figure 2.**
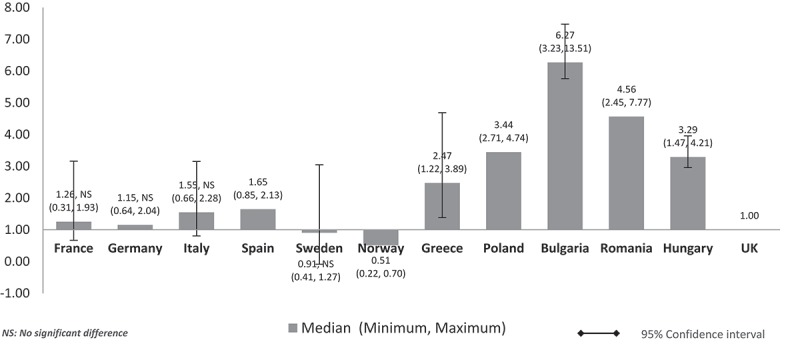
Relative cost ratios: adjusted using nominal GDP per capita (UK reference = 1).

When the costs were adjusted using GDP per capita, the lower GDP countries Bulgaria, Romania, Poland, and Hungary showed higher median costs than high GDP countries, and three to six times higher costs than the UK (Bulgaria: 6.27, Romania: 4.56, Poland: 3.44, Hungary: 3.29) (Figure 2). High GDP countries maintained minimal differences. Median costs in Spain (1.56), Italy (1.55), France (1.26), and Germany (1.15) were higher than in the UK. Sweden’s (0.91) and Norway’s (0.51) median costs were 9% and 49% cheaper than the UK, respectively. Norway retained the lowest cost ratio among all countries but with a wider gap (0.51) compared to the unadjusted costs in Figure 1 (0.87). Differences were significant for all except between the UK and France, Germany, Italy, and Sweden (high GDP countries).

**Figure 3.**
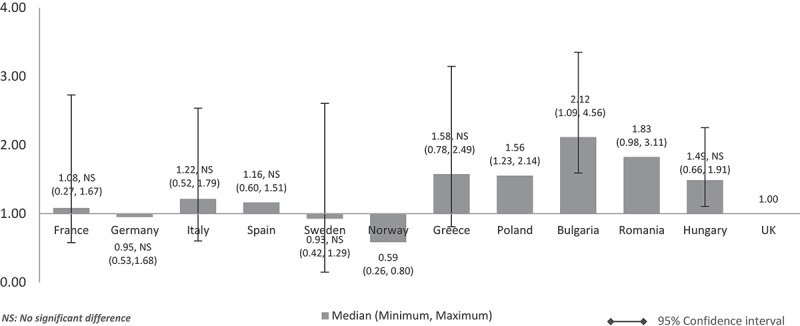
Relative cost ratios: adjusted using GDP in PPP per capita (UK reference = 1).

When the costs were adjusted using GDP in PPP per capita, the median costs in low GDP countries remained higher than in high-GDP countries (Figure 3). However, the cost differences between high GDP and low GDP countries were minimised compared to cost-adjusted figures using nominal GDP per capita. Median drug costs in Bulgaria, Romania, Polandm and Hungary were around one and a half to two times higher than the UK (Bulgaria: 2.12, Romania: 1.83, Poland: 1.56, Hungary: 1.49). Homogenisation of cost difference was also seen among high GDP countries when the costs are adjusted using GDP in PPP instead of nominal GDP per capita. The median costs in Spain (1.16), Italy (1.22), and France (1.08) were higher than the UK but to a lesser extent. Sweden (0.93) and Norway’s (0.59) median costs were still cheaper than the UK but to a lesser extent, now only at 7% from 9% and 41% from 49% cheaper than the UK respectively. Germany’s median cost was 5% cheaper than the UK (0.95) unlike in Figure 2. Norway remained with the lowest median costs. Differences were significant for all except between UK and France, Germany, Italy, Spain, Sweden, Greece, and Hungary.

**Figure 4.**
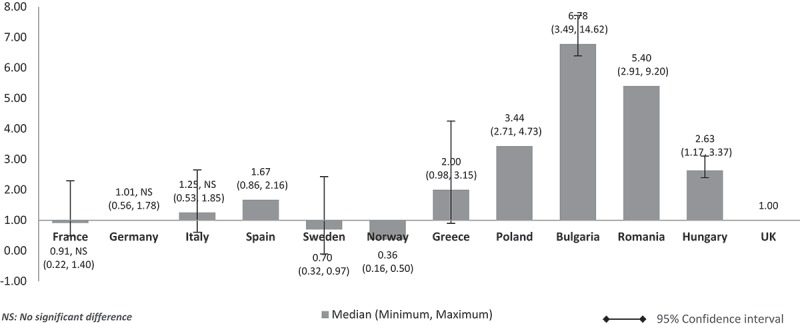
Relative cost ratios: adjusted using % GPD contributed by the government (UK reference = 1).

**Figure 5.**
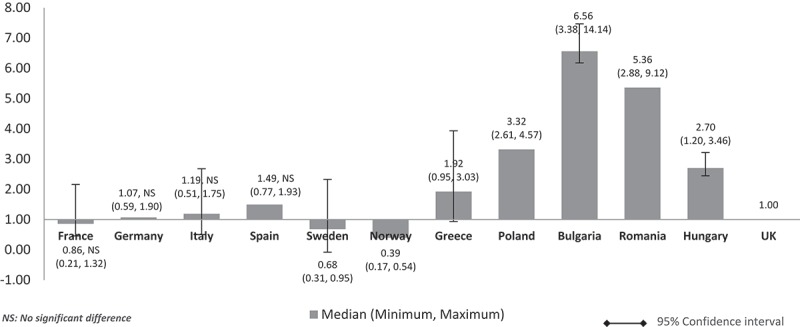
Relative cost ratios: adjusted using government budget per inhabitant (UK reference = 1).

When the costs were adjusted using GDP share contributed by the government (%) (Figure 4), the pattern was similar to Figure 2 (costs adjusted using nominal GDP per capita). The lower GDP countries Poland, Bulgaria, Romania, and Hungary showed higher median costs than in high GDP countries and at three to seven times more than the UK. Differences were significant for all except between the UK and France, Germany, and Italy. The same cost pattern was seen when the median costs were adjusted using government budget per inhabitant (Figure 5). Differences were significant for all except between UK and France, Germany, Italy, and Spain.

**Figure 6.**
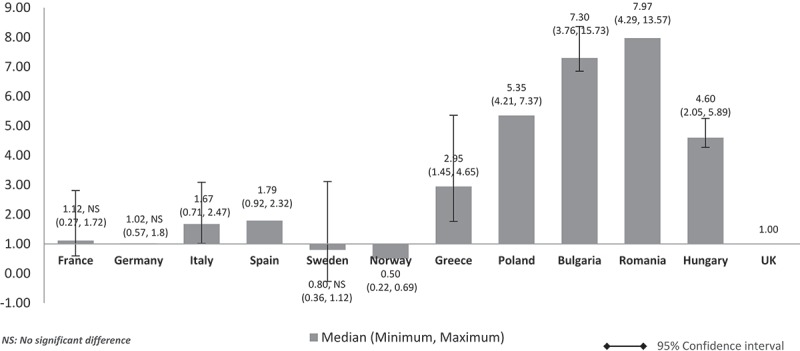
Relative cost ratios: adjusted using % GPD spent on healthcare (UK reference = 1).

Costs adjusted using GDP share spent on healthcare (%) (Figure 6) showed a similar pattern to Figures 2, 4, and 5 (costs adjusted using nominal GDP per capita, GDP share contributed by the government, government budget per inhabitant, respectively). The costs of orphan drugs in low GDP countries are higher compared to high GDP countries and are five to eight times higher than the UK. Differences were significant for all except between the UK and France, Germany, and Sweden.

**Figure 7.**
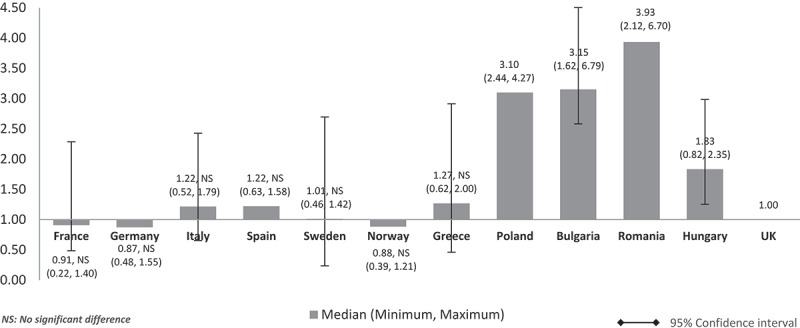
Relative cost ratios: adjusted using % GPD spent on pharmaceuticals (UK reference = 1).

Adjusted orphan drugs costs using GDP share spent on pharmaceuticals (%) (Figure 7) showed a pattern similar to when costs were adjusted using GDP in PPP per capita (Figure 3). The lower GDP countries Poland, Bulgaria, Romania, and Hungary showed higher costs than the costs in high GDP countries by a factor of around two. Differences were significant for all except between the UK and France, Germany, Italy, Spain, Sweden, Norway, and Greece.

**Figure 8.**
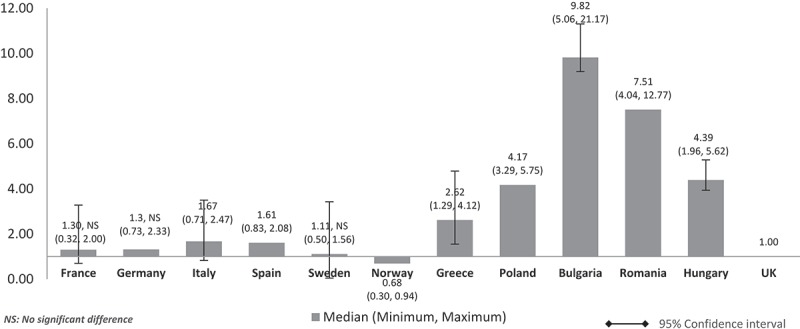
Relative cost ratios: adjusted using average annual salary per inhabitant (UK reference = 1).

When the costs were adjusted using average annual salary per inhabitant (Figure 8), the pattern and size of difference were similar to Figures 2, 4, 5 and 6 (costs adjusted using nominal GDP per capita, GDP share contributed by the government, government budget per inhabitant, GDP share spent on healthcare, respectively). The lower GDP countries Poland, Bulgaria, Romania, and Hungary showed higher costs, at four to nine times more than the UK, and higher than the costs in other high GDP countries. Differences were significant for all except between the UK and France, Germany, and Sweden.

## Discussion

When looking at orphan drugs costs based on list prices among the selected sample of EU countries, they appear to be very close. The lowest cost is seen in Norway which by far is not a low GDP country. When we adjusted costs using seven economic parameters (affordability variables) (Figures 2–8), the median annual treatment costs were consistently higher for low GDP countries than high GDP countries. This means that low GDP countries have to engage significantly more resources to provide orphan drug access to their population through the national healthcare system coverage.

Various studies in the literature have investigated the relevant issue of inequitable access to drugs between high GDP and low GDP countries in Europe [22–24]. Of note, these studies assessed innovative medicines that are able to offer patients better outcomes. Thus, equitable access was not only right but also imperative for patients. The 2016 IHE comparator report on patient access to cancer medicines in Europe presented unequal access to oncology treatments between low GDP/capita and high GDP/capita countries [22]. Eastern and southern Europe’s oncology drug sales were approximately a third of the sales in western Europe [22]. The same sales trend was seen in 2005 and in 2014 indicating a persistent trend [22]. In the field of infectious disease, in 2016 Iyengar S. et al. [23] assessed the affordability of the innovative Hepatitis C treatments sofosbuvir and ledspavir in 30 countries. Results show that central and eastern European countries had higher PPP-adjusted prices, indicating that low to median income countries may be paying relatively higher costs than economically stronger countries [23]. On the contrary, countries with high purchasing power had lower PPP-adjusted prices rendering higher affordability and better access to innovative treatments [23]. In 2012, Carone et al. noted in their report that drug prices adjusted using GDP per capita were higher in Poland, Romania, and Bulgaria as compared to Germany, Denmark, and Italy [25]. No study was found assessing the relative prices of orphan drugs. To the authors’ knowledge, this is the first EU study comparing orphan drug costs taking into consideration each country’s ability to pay.

### Economic parameters

Different economic parameters were used in this study to assess the orphan drug affordability of each country. GDP is commonly used to demonstrate the economic performance of a country or region. GDP in PPP factors in the purchasing power of the different currencies. The % of GDP contributed by the government gives us insight into how much of the GDP is contributed by the public system versus private institutions. This provides us with information when we analyse the % of GDP spent on healthcare and % of GDP spent on pharmaceuticals. If the contribution of the government to the GDP is significant and there is a significant % of GDP spent on healthcare or pharmaceuticals, this indirectly shows us that the National Health System has the willingness to spend on healthcare or pharmaceutical products. The % of GDP contributed by the government is also complemented by government budget per inhabitant which also shows us the ability to spend on individuals as part of the healthcare service. Lastly, as the scope of public funding for healthcare may not be comprehensive in all countries, out-of-pocket payment remains a reality. The average annual salary gives us an insight into patients’ ability to pay for pharmaceuticals themselves.

The majority of the economic parameters were gathered from the OECD database [17]. Per the OECD website [26],

PPPs are the rates of currency conversion that equalize the purchasing power of different currencies by eliminating the differences in price levels between countries. In their simplest form, PPPs are simply price relatives that show the ratio of the prices in national currencies of the same good or service in different countries.

This explains the homogenisation of cost difference among countries when the costs were adjusted using GDP in PPP per capita (Figure 3). Even so and however minimised, the median costs in low GDP countries remained higher than the median costs in high GDP countries.

The same minimal difference cost pattern was also seen when the costs were adjusted using GDP share spent on pharmaceuticals (Figure 7). The lower GDP countries Poland, Bulgaria, Romania, and Hungary showed higher relative costs than high GDP countries by only a factor of around two compared to three to six times when GDP per capita was used (Figure 2). This could be attributed to the 20% faster pace of annual increase in per capita spending on medicines in eastern Europe compared to western Europe [24]. In the past four decades, the central and eastern European countries increased their annual rate of spending on pharmaceuticals eight times faster than the EU-15 [24]. However, this does not directly translate to the citizen’s ability to purchase high-cost medicines in these countries. Drug spending was and is mostly from private funds and out-of-pocket expenditure is highly predominant [24]. With minimal public funding support, affordability issues still ensue. In Figure 8 (average annual salary per inhabitant), the lower GDP countries showed higher costs than high GDP countries by a factor of around three to nine. This means that an average individual will have to work up to nine times more (Bulgaria) in lower GDP countries in order to afford the same drug. Figure 4 (GDP share contributed by the government) and Figure 5 (government budget per inhabitant) show the lower per capita spending by the government in low GDP countries resulting in increased relative costs per patient, lower ability to pay, and thus in turn may lead to limited access to orphan drugs for rare diseases which lack alternative treatment.

### ERP implications

External reference pricing (ERP) has become the most common price setting measure for pharmaceuticals in EU member states. ERP, which is also known under different names such as external price referencing (EPR) or international price comparison/benchmarking, is defined as ‘the practice of using the price(s) of a medicine in one or several countries in order to derive a benchmark or reference price for the purposes of setting or negotiating the price of the product in a given country’ [27]. Thus, ERP as a price regulation tool should technically lead to price convergence in a region such as Europe where 29 of the 31 countries apply ERP (the UK and Sweden do not use ERP) [28]. Although no objective and comprehensive literature has been found assessing to what extent ERP is used in orphan drug pricing in Europe, our results show that ERP may have converged the absolute prices of orphan drugs but may have caused relative costs to differ as purchasing power is not taken into account in ERP price calculations and in the selection of reference countries [28]. Ex-factory price is the most commonly used reference in ERP [28] which in itself is limiting as it does not reflect the final price after confidential negotiations and Market Access Agreements between payers and manufacturers. High GDP countries such as the EU-5 have greater payer negotiation powers due to their size and revenue. These large and rich countries exercise their purchasing power to obtain confidential rebates that could be as high as 60%. Such rebates, being confidential, will not be integrated into ERP rules. Thus, lower GDP countries end up referencing inaccurately higher prices, adding burden to the already higher relative costs. Moreover, low GDP countries have low purchasing power and negotiation capacity thus will not obtain significant hidden discounts as in high GDP countries. From the authors’ experience, rebates do exist in lower GDP countries in the EU but to a lower magnitude than in the highest GDP countries. Manufacturers are also noted to use launch sequence strategies to avoid initially launching in countries with low price potential. Thus, they avoid the consequence that the low list price will inevitably be referenced by bigger markets. Launch may then be delayed in low GDP countries [28]. This further limits access to innovative and potentially life-saving treatments.

The use of ERP is reasonable in a region where parallel trade is allowed such as the EU. Price differentials based on purchasing power have repercussions. High price differences may lead to parallel imports from low-priced countries to higher-priced countries resulting in drug shortages in low-priced countries [25]. As such, similarity in absolute prices among neighbouring countries is a rational method to avoid such a scenario and thus ERP is continuously utilised by pharmaceutical companies in Europe to avoid parallel trade.

On the other hand, the perverse consequence of ERP is the acceleration of access and lowering of orphan drugs net prices in high GDP EU countries while delaying or preventing access and increasing net prices in low GDP countries. This unacceptable inequity is the outcome of setting ERP regulations in all countries. All regulations, even if justified, may have a perverse effect such as described here.

The European pricing policy is changing and new rules are expected as European cross-country initiatives on orphan drug procurement move forward [29]. Individual Member States initiatives are happening to address the high prices of orphan drugs. In November 2016, Bulgaria and Romania officially agreed on cross-border price negotiation and procurement of high-cost drugs [29,30]. In March 2017, the health ministers of the Visegrád 4 (V4) group of countries Poland, Czech Republic, Slovakia, and Hungary, as well as the health ministers of Lithuania and Croatia, signed a memorandum of understanding on co-operation in the area of ‘fair pricing’ for medicine [31]. For BeNeLuxA, a multilateral collaboration between the Netherlands and Belgium was established in April 2015 and has since expanded to include Luxembourg (September 2015) and Austria (June 2016) [29]. The initiative covers four areas of co-operation: (1) horizon-scanning databases to detect potentially significant orphan drugs in development; (2) joint research on the market and products; (3) combinations of two or more countries conducting health technology (HTA) evaluations; and (4) collective bargaining for select high-cost medicines (which can also encompass market access discussions and, in certain circumstances, Managed Entry Agreements) [29]. BeNeLuxA’s first joint medicine evaluation and pricing and reimbursement negotiation took place in May 2017 [32] when the Netherlands and Belgium concluded the first joint medicine evaluation and negotiation. Both countries adopted a negative reimbursement decision for the cystic fibrosis drug Orkambi (lumacaftor + ivacaftor) after three months of price negotiations with the US manufacturer Vertex. Lastly in South Europe, in May 2017, the health ministers of Malta, Cyprus, Greece, Italy, Spain, and Portugal signed the ‘Valletta Declaration’ concerning co-operation on the negotiation of drug prices in the Maltese capital of Valleta in connection with Malta’s EU presidency [33].

### The need for differential pricing

Considering that orphan drugs are known to be more expensive than non-orphan drugs [34], the non-affordability of low GDP countries is an urgent issue as unmet needs in these diseases are high and treatment options are limited or absent. Several discussions on differential pricing have been done in conferences and in the literature [35–39].

Differential pricing (DPR) ‘is based on the economic concept of price discrimination whereby prices of the same products are variedly set for different consumer groups in different geographical or socio-economic segments based on the income or purchasing power of those buyers’ [40]. DPR has been effectively used in vaccines, contraceptives, and anti-retroviral treatments globally.

Amidst the discussion on how differential pricing can offer solutions to the ongoing issue of inequitable access to drugs, implementation may be challenging. A concrete technical framework on how it will be implemented such as and not limited to the differentiation and grouping of countries based on income levels, harmonisation of value assessment, agreeing on the formula to be used for differential price calculations, and the appropriate management of parallel trade is needed for it to be an effective and sustainable solution [35–39]. Policy change, co-operation among member states, political will, and co-operation between the industry and the public healthcare systems are imperative to achieve these [35–40].

It has been possible to set prices for agricultural products across the EU with regulatory mechanisms to differentially compensate the impact on revenue in various geographical areas. There is no reason why a similar process could not be established for pharmaceutical drug pricing. As with food and nutrition, developing a differential pricing framework for a politically sensitive and complex area like health may be a long process [27]. Across Europe, the proportion of diagnosed patients can vary significantly due to differences in disease awareness and diagnostic tools. The excellence centres that treat orphan diseases are also not equal.

The authors are not proposing any specific methodology on how to implement the differentiation and grouping of countries and the subsequent setting of differential prices. Macroeconomists are the most appropriate people to choose the best economic criteria to measure a country’s ability to pay. This decision should be made at a European level. The European cross-country initiatives on orphan drug procurement discussed above are good platforms for discussing how differential pricing may be implemented.

Together with its strengths, weaknesses, opportunities, and threats, differential pricing, as a method complementary to other pricing tools such as ERP, may improve the affordability and accessibility of orphan drugs to vulnerable patients in lower GDP countries. The volume of products may be lower in low GDP countries due to higher prices and lower affordability. From a manufacturer’s perspective, differential pricing may pave the way for additional revenues through volume which may counter the lower price while maintaining the needed revenues to incentivise research and development from high GDP countries [39–42].

### Limitations of the study

The first limitation is that the basket of available products is not the same in all countries. It is probable that some countries may not reimburse expensive products. Hospital products may not have publicly listed prices and therefore were not available for analysis. The second limitation is that the net prices were not available and ex-factory prices were used for analysis. Very high rebates are regularly agreed with manufacturers in western European countries where they have higher power to negotiate prices as they are huge and important markets. However, in our previous study, we showed that there is a low difference in the median prices of similarly available orphan drugs in Europe [11].

It would have been good to illustrate the ERP calculation formula and the basket of reference countries used in orphan drug pricing at least for the lower GDP countries. However, no objective and comprehensive literature has been found assessing to what extent ERP is used in orphan drug pricing in Europe.

Although GDP contraction may have occurred at varied degrees per country after the 2008 financial crisis, the similarity of the patterns when costs were adjusted using GDP-related parameters and non-GDP parameters validate the finding that low GDP countries pay more for the same drugs compared to high GDP countries.

Lastly, price is not a good proxy for drug usage. As an example, obtaining a listed drug price may be relatively easy in the UK but it may not guarantee patient access and is only a formal step. However, it is logical that high price limits affordability and access especially in low-income countries. Further research on the relationship between orphan drug price and market share in value and volume will provide additional insight. There are however few resources available to compare the volumes to validate this reasoning.

## Conclusion

Individual orphan drug prices may vary widely across European countries but the median price differences are relatively minor. However, when the country’s ability to pay was taken into consideration by comparing relative costs adjusted using economic parameters, lower GDP countries pay relatively higher price for similarly available orphan drugs in Europe. Moreover, richer countries benefit more from substantial rebates and early access compared to poorer countries. Undisclosed rebates may exist in lower GDP countries but to a lesser extent than in higher GDP countries. This is the perverse consequence of ERP. Differential pricing, as complementary to other pricing tools such as ERP, may improve the affordability and accessibility of vulnerable patients to orphan drugs and, in general, to expensive and innovative products in lower GDP European countries.

## Supplementary Material

The_Perverse_impact_of_ERP_SUPP.docx

## Supplementary material

The supplementary material for this article can be accessed here.
